# Tumor attachment to Major intrahepatic vascular for Colorectal liver metastases

**DOI:** 10.1186/s12893-023-01971-2

**Published:** 2023-06-23

**Authors:** Wei Liu, Yong Cui, Xiao-Gang Wu, Feng-Lin Chen, Kun Wang, Ying-Shi Sun, Bao-Cai Xing

**Affiliations:** 1 Hepatopancreatobiliary Surgery Department I, Beijing Cancer Hospital and Institute, Key Laboratory of Carcinogenesis and Translational Research, Ministry of Education, Peking University School of Oncology, No. 52, Fu-Cheng-Lu Street, 100142 Beijing, China; 2 Department of Radiology, Peking University School of Oncology, Beijing Cancer Hospital and Institute, Key Laboratory of Carcinogenesis and Translational Research, Ministry of Education, No. 52, Fu-Cheng-Lu Street, 100142 Beijing, China

**Keywords:** Colorectal cancer, Hepatic metastasis, R1, Recurrence

## Abstract

**Background:**

Colorectal liver metastases attached major intrahepatic vessels has been considered to be a risk factor for survival outcome after liver resection. The present study aimed to clarify the outcomes of R1 surgery (margin < 1 mm) in CRLM patients, distinguishing parenchymal margin R1 and attached to major intrahepatic vessels R1.

**Methods:**

In present study, 283 CRLM patients who were evaluated to be attached to major intrahepatic vessels initially and underwent liver resection following preoperative chemotherapy. They were assigned to two following groups: R0 (*n* = 167), R1 parenchymal (*n* = 58) and R1 vascular (*n* = 58). The survival outcomes and local recurrence rates were analyzed in each group.

**Results:**

Overall, 3- and 5-year overall survival rates after liver resection were 53.0% and 38.2% (median overall survival 37 months). Five-year overall survival was higher in patients with R0 than parenchymal R1 (44.9%% vs. 26.3%, *p* = 0.009), whereas there was no significant difference from patients with vascular R1 (34.3%, *p* = 0.752). In the multivariable analysis, preoperative chemotherapy > 4 cycles, clinical risk score 3–5, RAS mutation, parenchymal R1 and CA199 > 100 IU/ml were identified as independent predictive factors of overall survival (*p* < 0.05). There was no significant difference for local recurrence among three groups.

**Conclusion:**

Parenchymal R1 resection was independent risk factor for CRLM. Vascular R1 surgery achieved survival outcomes equivalent to R0 resection. Non-anatomic liver resection for CRLM attached to intrahepatic vessels might be pursued to increase patient resectability by preoperative chemotherapy.

**Supplementary Information:**

The online version contains supplementary material available at 10.1186/s12893-023-01971-2.

## Introduction

Colorectal carcinoma is the third most common cancer worldwide [1]. Colorectal liver metastases (CRLM) will develop in half of patients in the course of disease and 25% will have synchronous CRLM at presentation. Liver resection (LR) has been considered to be only opportunity to cure the disease with 5-year overall survival (OS) ranging from 45 to 50% [2]. Preoperative chemotherapy was recommended to initially unresectable CRLM disease in conversional treatment. It was also administered to resectable patients as neoadjuvant chemotherapy to evaluate tumor behavior [3].

Anatomic liver resection always combined to remove major intrahepatic vessels and areas they supply or drained. With the efficiency increasing of surgical technique and modern chemotherapy, parenchymal‐sparing hepatectomy (PSH) has widely accept to be a standard surgical procedure for CRLM [4]. It is less invasive and leaves a more functional remnant liver [5]. While PSH might induce microscopically positive margin to preserve vessels by wedge liver resection that might be crucial for long-term survival [6]. Therefore, whether PSH is appropriate for CRLM still under debated in condition of major intrahepatic vessels attached.

The present study intended to assess whether preoperative chemotherapy improve resectability for CRLM attached major intrahepatic vessels by PSH. It also investigated to clarify the clinical relevance of R1 resection for CRLM with a focus on the distinction between tumor exposure along the transection plane (parenchymal R1) and CRLM initially attached major intrahepatic vessels (vascular R1). The outcomes of the two R1 procedures were compared with those of standard R0 resections in a per-patient and a per-resection area analysis.

## Materials and methods

### Patients

From January 2017 to December 2021, CRLM patients who were evaluated that tumor lesion attached to major intrahepatic vessels and underwent hepatic resection following preoperative chemotherapy at the Hepatopancreatobiliary Surgery Department I of Peking University Cancer Hospital were retrospectively reviewed.

### Study design

At pathology, a margin width ≥ 1 mm was classified as R0 resection, whereas a margin width < 1 mm was classified as R1 resection [7]. Patients with multiple liver resection were classified as R1 if at least one resection area had margin < 1 mm. R1 resections were included in vascular R1 (R1v) and parenchymal R1 (R1p). The R1v was defined that tumor exposed exclusively along the vessel [8]. R1p was defined that tumor exposed along the transection plane. The three groups, R0, R1v, and R1p were compared. Local recurrence was defined as any cut-edge recurrence diagnosed at the follow-up imaging (all the radiological images were reviewed). MR images were evaluated by two radiologists (Yong Cui and Qian Xing). The two radiologists adopted a consensus evaluation method, performed one-to-one correspondence before preoperative chemotherapy and liver resection. Per-patient and per-resection area analyses were performed. The present study was approved by the local ethics committee.

### Routine examination

Preoperative staging included carcinoembryonic antigen level, total colonoscopy, thoraci CT, abdominal contrast enhanced CT (CE-CT) and hepatic dynamic enhanced contrast MRI (DCE-MRI) with tissue-specific contrast agent. Follow-up was performed every 3 months and included carcinoembryonic antigen levels and abdominal ultrasonography, CT, or DCE-MRI.

### Inclusion and exclusion criteria

The inclusion criteria were: 1) liver metastasis was evaluated to be attached to major intrahepatic vessels; it was defined that CRLM attached first/second-order glissonean pedicles or hepatic veins within their last 4 cm before confluence into the inferior vena cava by MRI; 2) patients who received preoperative chemotherapy including neoadjuvant or conversional chemotherapy followed by liver resection; 3) there were no other simultaneous malignancies; 4) age 19 to 80 years; 5) an Eastern Co-operative Oncology Group (ECOG) performance status < 2; 6) patients with extrahepatic metastases only with resectable lung metastases. The exclusion criteria: 1) patients who underwent only ablation or palliative hepatic resection (R2); 2) patients were resected with both R1v and R1p were excluded.

### Surgery

The technical criteria of resectablitiy related to the liver remnant after resection: a) the anticipated ability to preserve two contiguous segments; b) the anticipated ability to preserve adequate vascular inflow, outflow and biliary drainage; c) the anticipated ability to preserve adequate future liver remnant volume (30% in normal liver and 40% in pretreated liver with chemotherapy) [9]. Any resection of three or more segments was considered a major hepatic resection. All the patients completed hepatic resection and primary tumor resection. Based on preoperative images, the tumor would be removed from intrahepatic major vessels as much as possible. If the vessel was testified to be invaded during operation, the anatomical liver resection was advocated. For patients whose lesions were suspicious attached to intrahepatic major vessels preoperatively, systematically IOUS to define the resection areas and determine to combine vascular resection.

### Statistical analysis

Patients were identified from a prospectively maintained database and retrospectively analyzed. Categorical variables were compared using the X^2^ or Fisher’s exact test. One continuous variable was analyzed and the Mann–Whitney U test was used. The Kaplan–Meier method was used to estimate survival probabilities, which were compared using the log-rank test. Disease free survival (DFS) was calculated as the time in months between the resection of primary tumor and metastases, and the diagnosis of recurrent disease. OS was calculated from the date of LR to the date of death or to the last follow-up contact. The date of the patient’s last contact was used as the end of follow-up in all censored patients, and no patient was lost to follow-up. Multivariate analysis was performed using a Cox proportional hazard model to identify independent prognostic factors of OS. Multivariate analysis was completed for factors with a p value in the univariate analysis. A *p* value < 0.05 was considered significant for all tests. A nomogram was created based on the results of the multivariable analysis. The predictive performance of the nomogram was assessed by evaluating the degree of discrimination with the C-index, plotting Kaplan–Meier curves over the quartiles of the nomogram-predicted score and examining calibration plots with bootstrapped samples.

## Result

Overall, 1,220 consecutive patients undergoing a first LR for CRLM in the study period were considered. Finally, 283 patients with 1,752 resection areas were analyzed. Patient characteristics are summarized in Table 1. In the whole series, 116 patients (41.0%) had R1 resection, including 58 (50.0%) with R1p and 58 (50.0%) with R1v. R1p had a higher rate of bilobar location while R1v patients had a higher rate of RAS wild type. R0, R1v, and R1p patients had similar morbidity and blood loss volume. The R1p group had a similar rate of liver-only recurrences (27 in the R1v and 34 in the R1p groups, *p* = 0.618). non anatomical resection (NAR) had a similar rate of recurrence in anatomic resection (AR) (18 vs. 9, *p* = 0.442). The local recurrence rate was similar in glissonean than hepatic vein (16 vs. 11, *p* = 0.737) (Table 2).

**Table 1:** Demographic and clinical characteristics of patients

	R0(*n* = 167)	R1V(*n* = 58)	R1P(*n* = 58)	*p*
Age(years)	56.1 ± 9.5	58.1 ± 8.1	58.8 ± 9.7	0.113
Sex(M)	108(64.7%)	43(74.1%)	37(63.8%)	0.376
Primary tumor				
Rectal/Colon	62/105	20/38	21/37	0.937
Right/Left	27/140	8/50	11/47	0.751
T1-2	11(6.6%)	3(5.2%)	5(8.6%)	0.755
T3-4	156(93.4%)	55(94.8%)	53(91.4%)	
Primary Node				
(-)	40(24.0%)	22(37.9%)	17(29.3%)	0.119
( +)	127(76.0%)	36(62.1%)	41(70.7%)	
Liver metastases				
Synchronous	151(90.4%)	51(87.9%)	54(93.1%)	0.638
Metachronous	16(9.6%)	7(12.1%)	4(6.9%)	
No. of metastases (median)	4(1–20)	4(1–20)		
Single metastases	26(15.6%)	11(19.0%)	4(6.9%)	0.15
Multiple metastases	141(84.4%)	47(81.0%)	54(93.1%)	
Tumor size				
> 50 mm	25(15.0%)	15(25.9%)	8(13.8%)	0.126
≤ 50 mm	142(85.0%)	43(74.1%)	50(86.2%)	
Unilobar	49(29.3%)	16(27.6%)	6(10.3%)	0.014
Bilobar	118(70.7%)	42(72.4%)	52(89.7%)	
Extrahepatic disease	35(21.0%)	5(8.6%)	13(22.4%)	0.084
CEA	26.64 ± 63.50	33.04 ± 79.02	29.53 ± 70.16	0.697
CA199	100.74 ± 38.68	46.27 ± 66.61	94.41 ± 52.16	0.435
CRS(0–2)				
CRS(3–5)				
Preoperative chemotherapy				
Neoadjuvant chemotherapy	77(46.1%)	28(48.3%)	19(32.8%)	0.15
Conversional chemotherapy	90(53.9%)	30(51.7%)	39(67.2%)	
Oxaliplatin	60(35.9%)	15(25.9%)	26(44.8%)	0.328
	R0(*n* = 167)	R1V(*n* = 58)	R1P(*n* = 58)	p
Irinotecan	52(31.1%)	20(34.5%)	15(25.9%)	
Both	55(33.0%)	23(39.6%)	17(29.3%)	
Ass bevacizumab	80(47.9%)	28(48.3%)	26(44.8%)	0.367
Ass cetuximab	67(40.1%)	24(41.4%)	22(37.9%)	
Both	3(1.8%)	0(0%)	4(6.9%)	
None	17(10.2%)	6(10.3%)	6(10.3%)	
Number of lines > 1	40(24.0%)	21(36.2%)	16(27.6%)	0.195
Number of cycles	5(2–23)	5(2–16)	5(2–14)	0.664
Response				0.963
PR	100(59.9%)	33(56.9%)	37(63.8%)	
SD	64(38.3%)	24(41.4%)	20(34.5%)	
PD	3(1.8%)	1(1.7%)	1(1.7%)	
Adjuvant chemo	137(82.0%)	47(81.0%)	44(75.9%)	0.530
RAS mutation	80(47.9%)	17(29.3%)	25(43.1%)	0.048
Before preoperative chemo				
Tumor attachment of vessels	219	103		
After preoperative chemo				
Anatomic resection	58(34.7%)	15(25.9%)	43(74.1%)	0.445
PSH	109(65.3%)	43(74.1%)	15(25.9%)	
Blood loss(ml)	244.0 ± 66.1	278.3 ± 20.5	306.0 ± 62.6	0.098
Major complication				
0-II	160	56	55	0.133
III-IV	7	2	3	
Blood transfusion	9	1	6	0.129

**Table 2:** Local recurrence after liver resection

	*n*	Local recurrence	Extrahepatic recurrence	*p*
Total				0.168
R0 Group	167	79	34	
R1v Group	58	27	18	
R1p Group	58	34	9	
R1v Group	58			0.737
Glissonean	32	16	10	
Hepatic Vein	26	11	8	
R1v Group				0.442
LR with vessels	15	9	3	
LR without vessels	43	18	15	

### Survival analysis

**Fig. 1:**
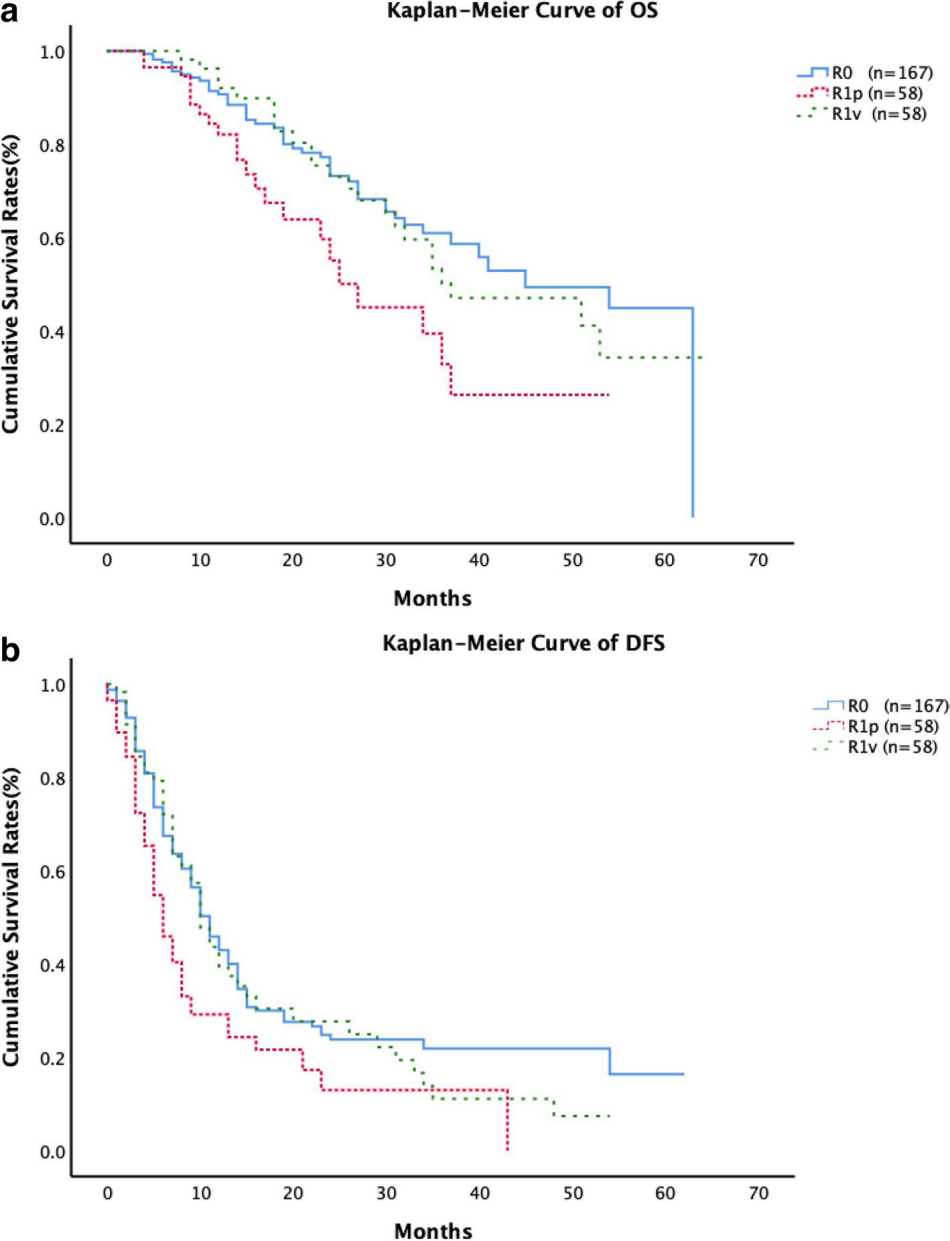
**a** The OS of R0, R1v and R1p was showed by Kaplan–Meier Curve, respectively. **b** The DFS of R0, R1v and R1p was showed by Kaplan–Meier Curve, respectively

The median followed up was 27 months since the first recurrence (95% CI: 23–30 months). Overall, 3- and 5-year OS rates after liver resection were 53.0% and 38.2% (median OS 37 months). Five-year OS was higher in patients with R0 than R1p (44.9%% vs. 26.3%, *p* = 0.009), whereas there was no significant difference from patients with R1v (34.3%, *p* = 0.752; Fig. 1a). Moreover, 3- and 5-year DFS rates after liver resection were 17.5% and 10.4% (median DFS 10 months). Three-year DFS was higher in patients with R0 than R1p (21.8%% vs. 13.0%, *p* = 0.004), whereas there was no significant difference from patients with R1v (11.1%, *p* = 0.612; Fig. 1b).

In the univariable analysis, including CRS 3–5, RAS mutation, R1v resection, preoperative chemotherapy > 4 cycles, distribution bilobar and CA199 > 100 IU/ml were identified as independent predictive factors of OS (*p* < 0.05). In the multivariable analysis, preoperative chemotherapy > 4 cycles, CRS 3–5, RAS mutation, R1p and CA199 > 100 IU/ml were identified as independent predictive factors of OS (*p* < 0.05) (Table 3).

**Table 3:** Univariable and multivariable analysis of factors associated with OS

	**Univariable analysis**			**Multivariable analysis**		
	**HR**	**95%**	***P* value**	**HR**	**95%**	***P* value**
Age						
> 70	Ref					
≤ 70	1.363	0.710–2.616	0.352			
Gender						
Male	Ref					
Female	1.183	0.782–1.791	0.426			
Primary T stage						
1–2	Ref					
3–4	0.651	0.328–1.294	0.651			
Primary N stage						
N0	Ref					
N1-2	1.384	0.894–2.142	0.145			
Location tumor						
Colon	Ref					
Rectum	1.091	0.737–1.615	0.663			
Primary tumor location						
Left	Ref					
Right	1.433	0.896–2.293	0.133			
Disease free interval						
> 12 month	Ref					
≤ 12 month	1.419	0.756–2.662	0.474			
CEA						
≤ 200	Ref					
> 200	1.448	0.587–3.571	0.421			
CA199						
> 100	Ref			Ref		
≤ 100	0.451	0.284–0.718	0.001	0.485	0.300–0.785	0.003
Tumor size						
≤ 5 cm	Ref					
> 5 cm	1.196	0.727–1.965	0.481			
Tumor no						
≤ 1	Ref					
> 1	0.613	0.328–1.146	0.126			
Neoadjuvant						
No	Ref					
Yes	0.677	0.457–1.003	0.052			
Ras status						
Wild	Ref			Ref		
Mutation	0.562	0.383–0.842	0.003	0.554	0.372–0.825	0.004
Hepatic resection						
Minor	Ref					
Major	1.072	0.841–1.297	0.114			
CRS						
0–2	Ref			Ref		
3–5	0.486	0.300–0.787	0.003	0.594	0.355–0.996	0.048
Margin status						
R0	Ref					
R1V	1.074	0.661–1.744	0.112			
R1P	2.039	1.155–3.599	0.014			
Line						
Line = 1	Ref					
Line > 1	1.268	0.831–1.934	0.271			
Cycles						
≤ 4	Ref			Ref		
> 4	0.528	0.352–0.790	0.002	0.533	0.352–0.808	0.003
Response						
PR	Ref					
SD	0.776	0.689–1.227	0.442			
PD	0.812	0.542–1.361	0.531			
Distribution						
Unilobar	Ref			Ref		
Bilobar	0.497	0.307–0.805	0.005	0.756	0.445–1.238	0.300
Complication						
Minor	Ref					
Major	1.563	0.914–7.407	0.442			
Adjuvant chemotherapy						
No	Ref					
Yes	1.114	0.689–1.437	0.267			

### Creation of a prognostic nomogram

**Fig. 2:**
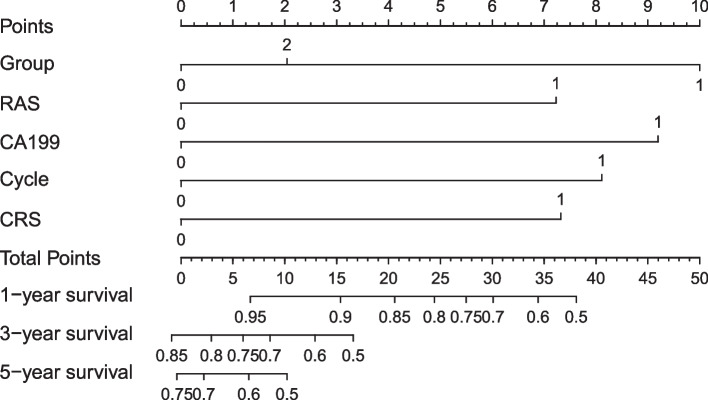
Colorectal liver metastasis Nomogram for OS

**Fig. 3:**
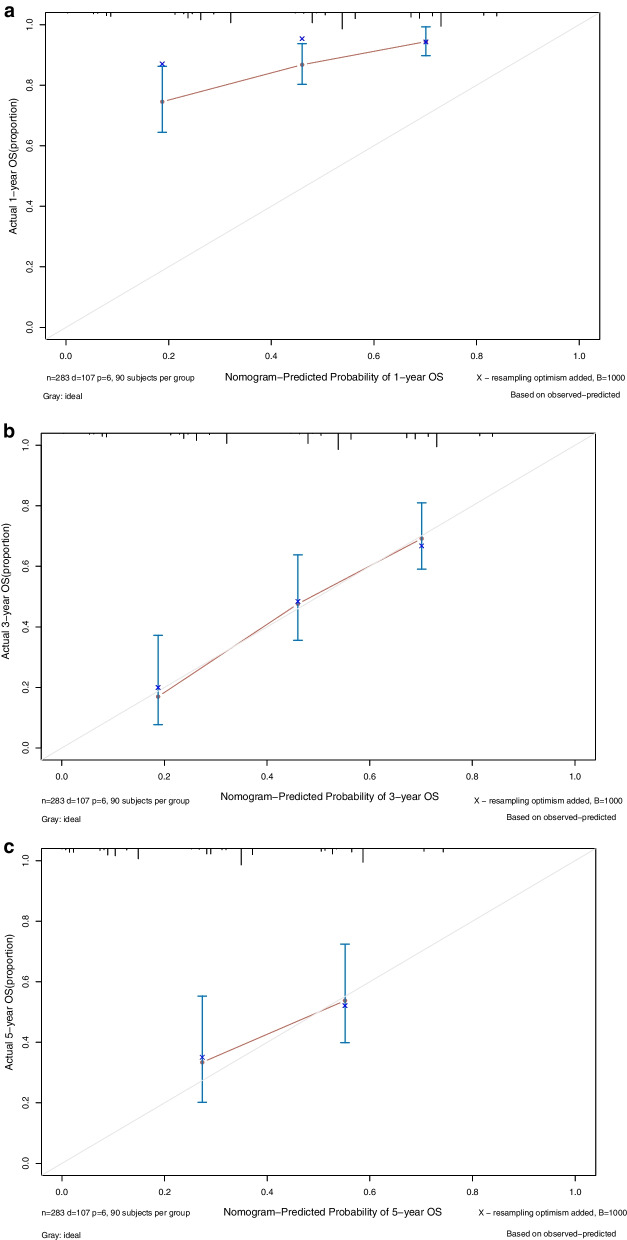
**a** The predicting OS at 1-year by calibration curve. **b** The predicting OS at 3-year by calibration curve. **c** The predicting OS at 5-year by calibration curve

A prognostic nomogram for OS after hepatectomy with point scales for the above five factors was constructed subsequently (Fig. 2). Based on the multivariable Cox model, these factors were assigned a specific score as follows: R1p, 10’; cycle > 4, 8’; RAS mutation, 7’; CA199 > 100, 9’; CRS, 7’ (Supplementary Table). The sum of the scores for each variable was plotted on the total points axis (left side), and the estimated probabilities of survival at 1, 3 and 5 years were obtained by drawing a line horizontally from the plotted total points axis straight to the survival axis (right side). Total points for the scores ranged from 0 to 41, and the C-statistic for OS prediction was 0.70. A calibration plot for the probability of survival at 1, 3 and 5 years demonstrated good calibration between the prediction by the nomogram and the actual observation (Fig. 3).

## Discussion

Liver resection is crucial for long-term survival. However, the R1v resection was still controversial with the effects of preserving major intrahepatic vessel contact with the CRLM. Therefore, we analyzed the impact of the surgical margin and vessel preservation on oncological outcomes. The present study indicated that R1 resections of CRLM were sufficient for local recurrence control, and that preserving hepatic vein contact with tumors is acceptable. Five-year OS was not significant difference in patients with R0 than R1v (44.9%% vs. 34.3%, *p* = 0.752).

AR has been advocated to liver resection for HCC in last decades [10]. Assuming a margin negative resection can be achieved, some authors also have suggested that a more aggressive resection strategy may be required for CRLM patients. Actually, similar survival outcomes can be achieved with both AR and NAR [11]. More aggressive surgical management in the setting of multidisciplinary patient care has been associated with a shift in focus to the remnant liver rather than the volume of tumor present. With an increased emphasis on preserving the remnant liver, there has been an increased interest in parenchymal versus non-parenchymal-sparing operations for CRLM. Regarding long-term OS, the majority of studies did not demonstrate a difference in OS among patients undergoing PSH versus AR for CRLM. When assessing OS in aggregate, there was no difference whether resection of CRLM was performed with PSH (5-year OS: mean 44.7%, range 29–62%) or AR (5-year OS: mean 44.6%, range 27–64%) (*p* = 0.97) [11]. It was consistent with hepatocellular carcinoma that extensive liver resection was not recommended even patients with portal vein thrombosis [12].

Surgical margin status is a technical, operative factor that has also been traditionally associated with long-term prognosis [13]. Historically, compared with R0 resections, R1 resections elevated risk of recurrence and significantly lower survival rates. However, the need to achieve an even R0 margin in the new era of modern chemotherapy has been recently challenged. Perioperative chemotherapy has been adopted routinely since the publication of the EORTC trial [14]. Preoperative chemotherapy might converse unresectable patients to hepatic resection and highly select candidates to surgery in resectable patients [15]. It was believed that increasingly efficient chemotherapy may have changed the long-term outcome after a R1 resection, especially in patients with advanced metastatic disease [6, 16]. A series of studies have testified and showed benefit of modern chemotherapy on R1 resection that there were no negative impacts in survival rates, particularly in patients with optimal morphological or major histopathological responses [17].

In the literature, as in the present series, local recurrence was rarely the only metastatic site and, whenever isolated, was resectable in most patients [18]. However, patients receiving R1p resection had twofold lower OS in comparison with the R0 group. This prognostic difference is well known in the literature [18]. It is unclear if R1p per se drives prognosis or if it is a surrogate of aggressive tumor biology, but should be avoided whenever possible. The long-term outcome of R1v resection remains to be clarified. These patients had higher rates of multiple and bilobar disease and of preoperative extrahepatic disease, which corresponded to a higher postoperative extrahepatic recurrence rate and early cancer-related mortality. Nevertheless, the intrahepatic recurrence rate and the long-term survival of the R1v group were similar to those of the R0 group, strongly suggesting the oncological adequacy of vascular detachment.

If a predicted positive surgical margin after resection is no longer an absolute contraindication to surgery for treating advanced and aggressive liver metastases [19], tumor size reduction by > 60% could permit resection that preserves the vessel showing attachment, without vascular resection or reconstruction. In such situations, liver metastases attached to or invading major intrahepatic vessels seemed not easily separable by treatment, even with a regimen including monoclonal antibodies [20, 21]. Extent of tumor attachment to the vessels and deformity of the vessels on CT were reported to be useful indications for concomitant liver and vessel resection, focusing on hepatic caval invasion of the liver tumors [22].

### Limitation

Firstly, the present study analyzed CRLM attached intrahepatic major vessel retrospectively by clinical risk factors without preoperative radiological information. Secondly, the nomogram is not fully accurate because the prediction was calculated based on the statistical significance within the collected factors. It still needs external validation. Finally, this was an observation cohort study and sample size limited.

## Conclusion

In conclusion, R1 resections for CRLM occurring apart from vessels can achieve good local control, regardless of the surgical margin width. When CRLM are in contact with the major intrahepatic vessels, the wedge resection also improves long-term survival with low recurrence.

## Supplementary Information

**Additional file 1: Supplementary Table. **Prognostic factor points for CRLM patients with tumor attached vessels. 

**Additional file 2: Supplementary Figure 1. **The images of a 50 years old CRLM patients with tumor attached vessels. **Supplementary** **Figure 2.** The images of a 71 years old CRLM patients with tumor attached vessels.

## Data Availability

The datasets generated and/or analyzed during the current study are not publicly available due to protecting individual patient privacy but are available from the corresponding author on reasonable request.

## Abbreviation

CRLM: Colorectal liver metastases
RFA: Radiofrequency ablation
OS: Overall survival
DFS: Disease free survival
MDT: Multidisciplinary team
CEA: Carcinoembryonic antigen
HR: Hazard ratio
MRI: Magnetic resonance imaging
CT: Computed tomography
IOUS: Intraoperative Ultrasound
LR: Liver resection
AR: Anatomical resection
NAR: Non- anatomical resection
CRS: Clinical risk score
PSH: Parenchymal‐sparing hepatectomy

## References

[1] Torre LA, Bray F, Siegel RL, Ferlay J, Lortet-Tieulent J, Jemal A (2015). Global cancer statistics, 2012. CA Cancer J Clin.

[2] Adam R, Kitano Y (2019). Multidisciplinary approach of liver metastases from colorectal cancer. Ann Gastroenterol Surg.

[3] Nierop PM, Hoppener DJ, Buisman FE, van der Stok EP, Galjart B, Balachandran VP, Jarnagin WR, Kingham TP, Shia J, Mauer M (2022). Preoperative systemic chemotherapy alters the histopathological growth patterns of colorectal liver metastases. J Pathol Clin Res.

[4] Torzilli G, Vigano L, Gatti A, Costa G, Cimino M, Procopio F, Donadon M, Del Fabbro D (2017). Twelve-year experience of "radical but conservative" liver surgery for colorectal metastases: impact on surgical practice and oncologic efficacy. HPB (Oxford).

[5] Spelt L, Ansari D, Swanling M, Holka P, Andersson R (2018). Parenchyma-sparing hepatectomy (PSH) versus non-PSH for bilobar liver metastases of colorectal cancer. Ann Gastroenterol.

[6] Tanaka K, Kumamoto T, Nojiri K, Takeda K, Endo I (2012). Postchemotherapy histological analysis of major intrahepatic vessels for reversal of attachment or invasion by colorectal liver metastases. Cancer.

[7] de Haas RJ, Wicherts DA, Flores E, Azoulay D, Castaing D, Adam R (2008). R1 resection by necessity for colorectal liver metastases: is it still a contraindication to surgery?. Ann Surg.

[8] Torzilli G, Montorsi M, Donadon M, Palmisano A, Del Fabbro D, Gambetti A, Olivari N, Makuuchi M (2005). "Radical but conservative" is the main goal for ultrasonography-guided liver resection: prospective validation of this approach. J Am Coll Surg.

[9] Adams RB, Aloia TA, Loyer E, Pawlik TM, Taouli B, Vauthey JN, Americas Hepato-Pancreato-Biliary A (2013). Society of Surgical O, Society for Surgery of the Alimentary T: Selection for hepatic resection of colorectal liver metastases: expert consensus statement. HPB (Oxford).

[10] Minagawa M, Mise Y, Omichi K, Ichida H, Mizuno T, Yoshioka R, Imamura H, Yanagisawa N, Inoue Y, Takahashi Y (2022). Anatomic Resection for Hepatocellular Carcinoma: Prognostic Impact Assessed from Recurrence Treatment. Ann Surg Oncol.

[11] Moris D, Ronnekleiv-Kelly S, Rahnemai-Azar AA, Felekouras E, Dillhoff M, Schmidt C, Pawlik TM (2017). Parenchymal-Sparing Versus Anatomic Liver Resection for Colorectal Liver Metastases: a Systematic Review. J Gastrointest Surg.

[12] Sena G, Paglione D, Gallo G, Goglia M, Osso M, Nardo B (2022). Surgical Resection of a Recurrent Hepatocellular Carcinoma with Portal Vein Thrombosis: Is It a Good Treatment Option? A Case Report and Systematic Review of the Literature. J Clin Med..

[13] Margonis GA, Sergentanis TN, Ntanasis-Stathopoulos I, Andreatos N, Tzanninis IG, Sasaki K, Psaltopoulou T, Wang J, Buettner S, Papalois Alpha E (2018). Impact of Surgical Margin Width on Recurrence and Overall Survival Following R0 Hepatic Resection of Colorectal Metastases: A Systematic Review and Meta-analysis. Ann Surg.

[14] Nordlinger B, Sorbye H, Glimelius B, Poston GJ, Schlag PM, Rougier P, Bechstein WO, Primrose JN, Walpole ET, Finch-Jones M (2013). Perioperative FOLFOX4 chemotherapy and surgery versus surgery alone for resectable liver metastases from colorectal cancer (EORTC 40983): long-term results of a randomised, controlled, phase 3 trial. Lancet Oncol.

[15] Nasti G, Ottaiano A, Berretta M, Delrio P, Izzo F, Cassata A, Romano C, Facchini G, Scala D, Mastro A (2010). Pre-operative chemotherapy for colorectal cancer liver metastases: an update of recent clinical trials. Cancer Chemother Pharmacol.

[16] Margonis GA, Kreis ME, Wang JJ, Kamphues C, Wolfgang CL, Weiss MJ (2020). Impact and clinical usefulness of genetic data in the surgical management of colorectal cancer liver metastasis: a narrative review. Hepatobiliary Surg Nutr.

[17] Protic M, Krsmanovic O, Solajic N, Kukic B, Nikolic I, Bogdanovic B, Radovanovic Z, Kresoja M, Mannion C, Man YG (2021). Prospective Non-Randomized Study of Intraoperative Assessment of Surgical Resection Margin of Colo-Rectal Liver Metastases. J Cancer.

[18] Poultsides GA, Schulick RD, Pawlik TM (2010). Hepatic resection for colorectal metastases: the impact of surgical margin status on outcome. HPB (Oxford).

[19] Tanaka K, Nojiri K, Kumamoto T, Takeda K, Endo I (2011). R1 resection for aggressive or advanced colorectal liver metastases is justified in combination with effective prehepatectomy chemotherapy. Eur J Surg Oncol.

[20] Hiroyoshi J, Arita J, Gonoi W, Akamatsu N, Kaneko J, Hasegawa K (2019). Significance of Glisson's capsule invasion in patients with colorectal liver metastases undergoing resection. Am J Surg.

[21] Vigano L, Procopio F, Cimino MM, Donadon M, Gatti A, Costa G, Del Fabbro D, Torzilli G (2016). Is Tumor Detachment from Vascular Structures Equivalent to R0 Resection in Surgery for Colorectal Liver Metastases?. An Observational Cohort Ann Surg Oncol.

[22] Hashimoto T, Minagawa M, Aoki T, Hasegawa K, Sano K, Imamura H, Sugawara Y, Makuuchi M, Kokudo N (2008). Caval invasion by liver tumor is limited. J Am Coll Surg.

