# Supplementary material for: Tumor attachment to Major intrahepatic vascular for Colorectal liver metastases
Source: BMC Surg. 2023 Jun 23;23:169. doi: 10.1186/s12893-023-01971-2 (PMC10290376; doi:10.1186/s12893-023-01971-2)
Supplement: Supplementary file 1 — Additional file 1: Supplementary Table. Prognostic factor points for CRLM patients with tumor attached vessels. [file 12893_2023_1971_MOESM1_ESM.docx]

**Supplementary Table 1. Specific score of risk factors**

| **Factor** | **Score** |
| --- | --- |
| Margin status |  |
| R0 | 0 |
| Parenchymal R1 | 10 |
| CRS |  |
| 0-2 | 0 |
| 3-5 | 7 |
| RAS status |  |
| Wild type | 0 |
| Mutation type | 7 |
| CA199 |  |
| ≤100 | 0 |
| >100 | 9 |
| Cycles |  |
| ≤4 | 0 |
| >4 | 8 |
