# Supplementary material for: Tumor attachment to Major intrahepatic vascular for Colorectal liver metastases
Source: BMC Surg. 2023 Jun 23;23:169. doi: 10.1186/s12893-023-01971-2 (PMC10290376; doi:10.1186/s12893-023-01971-2)
Supplement: Supplementary file 2 — Additional file 2: Supplementary Figure 1. The images of a 50 years old CRLM patients with tumor attached vessels. Supplementary Figure 2. The images of a 71 years old CRLM patients with tumor attached vessels. [file 12893_2023_1971_MOESM2_ESM.docx]

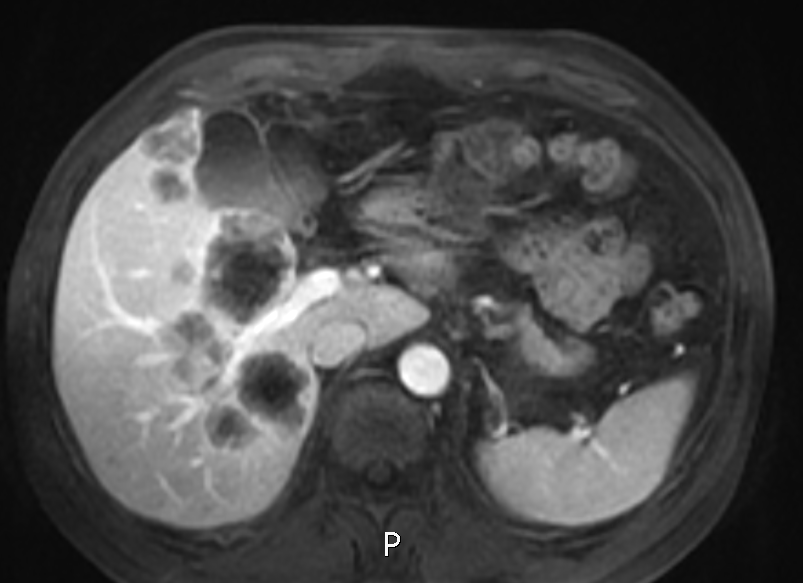


A


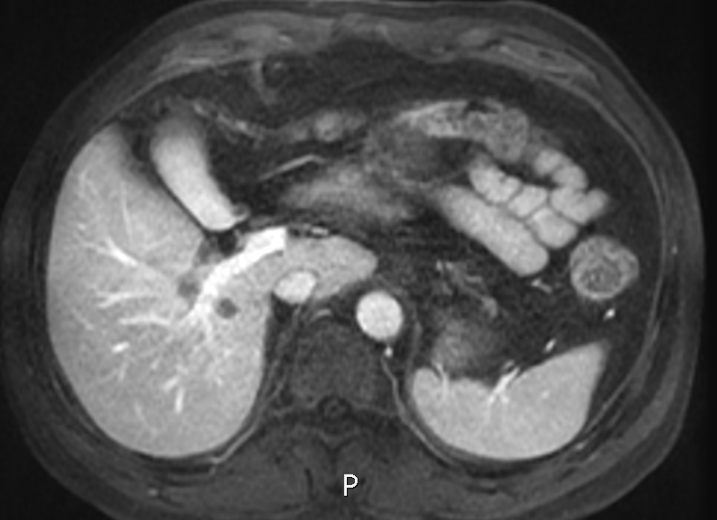


B


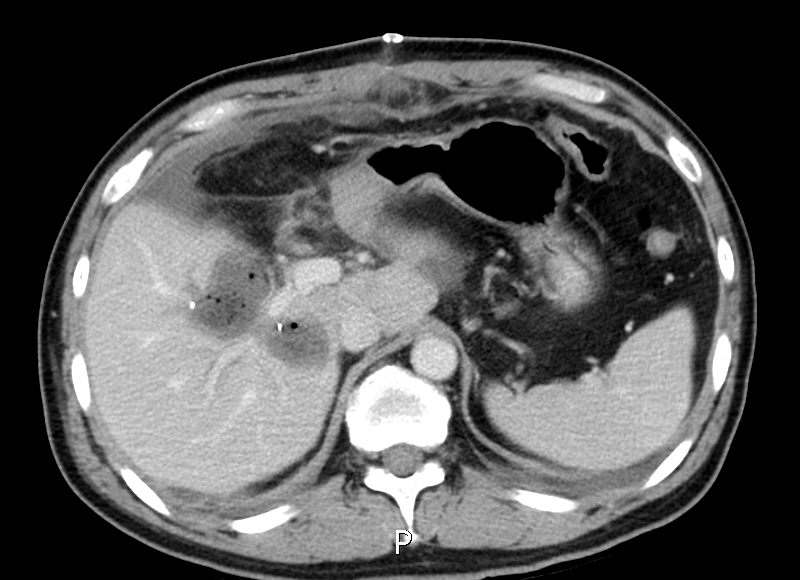


C

Supplementary Figure 1. A 50-year old man with sigmoid colon cancer. (A) Hepatic magnetic resonance (MR) scan before initiation of chemotherapy shows liver metastases is attached to the right posterior branch of Glisson’s pedicle. (B) MR scan shows the liver metastases remained attached to the right posterior branch 6 months after initiation of chemotherapy. (C) Computed tomography (CT) scan after the resection of liver metastases shows the right posterior branch of Glisson’s pedicle was preserved.


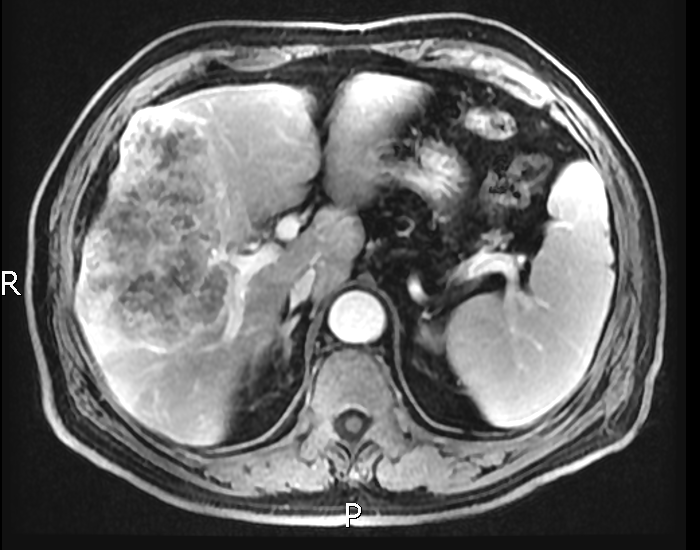


A


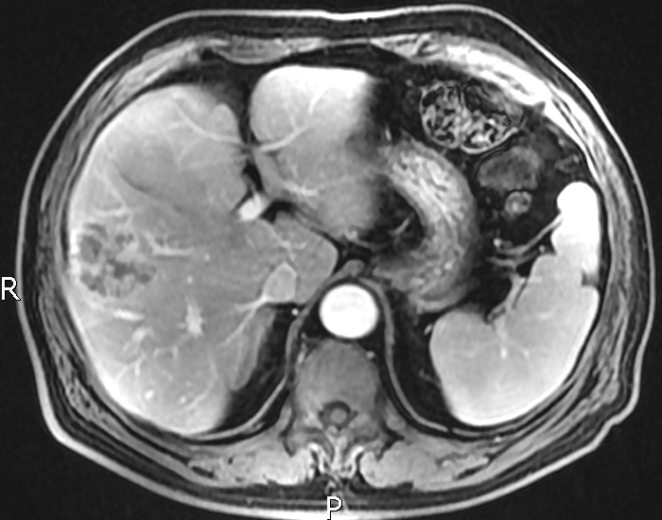


B


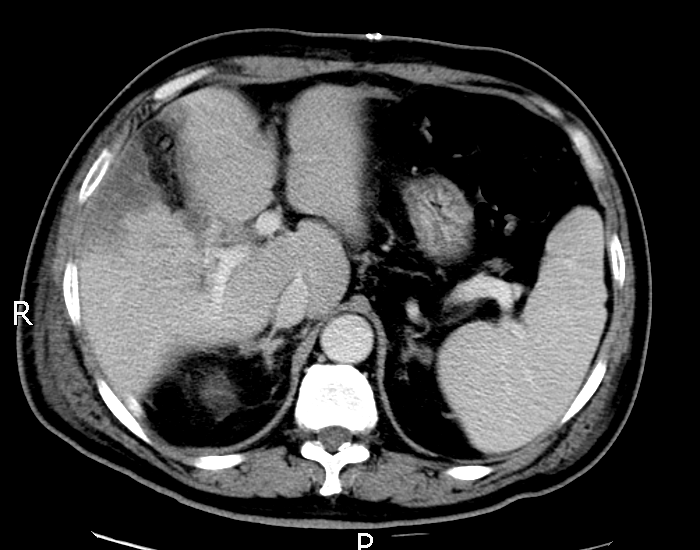


C

Supplementary Figure 2. A 71-year old man with sigmoid colon. (A) Hepatic magnetic resonance (MR) scan before initiation of chemotherapy shows liver metastases is attached to the right anterior branch of Glisson’s pedicle. (B) MR scan shows the liver metastases remained attached to the right anterior branch 3 months after initiation of chemotherapy. (C) Computed tomography (CT) scan after the resection of liver metastases shows the right anterior branch of Glisson’s pedicle was removed.
